# Experimental evidence for beneficial effects of projected climate change on hibernating amphibians

**DOI:** 10.1038/srep26754

**Published:** 2016-05-27

**Authors:** Bálint Üveges, Katharina Mahr, Márk Szederkényi, Veronika Bókony, Herbert Hoi, Attila Hettyey

**Affiliations:** 1 Konrad Lorenz Institute of Ethology, Department of Integrative Biology and Evolution, University of Veterinary Medicine Vienna, Savoyenstraße 1A, 1160 Vienna, Austria; 2 Lendület Evolutionary Ecology Research Group, Plant Protection Institute, Centre for Agricultural Research, Hungarian Academy of Sciences, Herman Ottó út 15, 1022 Budapest, Hungary

## Abstract

Amphibians are the most threatened vertebrates today, experiencing worldwide declines. In recent years considerable effort was invested in exposing the causes of these declines. Climate change has been identified as such a cause; however, the expectable effects of predicted milder, shorter winters on hibernation success of temperate-zone Amphibians have remained controversial, mainly due to a lack of controlled experimental studies. Here we present a laboratory experiment, testing the effects of simulated climate change on hibernating juvenile common toads (*Bufo bufo*). We simulated hibernation conditions by exposing toadlets to current (1.5 °C) or elevated (4.5 °C) hibernation temperatures in combination with current (91 days) or shortened (61 days) hibernation length. We found that a shorter winter and milder hibernation temperature increased survival of toads during hibernation. Furthermore, the increase in temperature and shortening of the cold period had a synergistic positive effect on body mass change during hibernation. Consequently, while climate change may pose severe challenges for amphibians of the temperate zone during their activity period, the negative effects may be dampened by shorter and milder winters experienced during hibernation.

Amphibians represent the most threatened vertebrate class today, with more than 42% of amphibian species being in decline^1^. Since the 1990s, the spatial and temporal patterns and the potential causes of amphibian declines attracted a large body of conservation-oriented research (for reviews see^1^,^2^,^3^). It became clear that not all species and populations are equally prone to decline, with tropical species being the most susceptible^1^,^2^,^4^,^5^, but population declines also happening in temperate regions^5^,^6^,^7^,^8^.

Climate change and its interactions with other factors have been recently identified as important contributors to the amphibian diversity crisis^4^,^9^,^10^,^11^,^12^,^13^. Because of their ectothermic nature, highly permeable skin, and complex life cycles, amphibians strongly depend on specific temperature ranges, moisture, and precipitation^1^,^14^,^15^, factors which are fundamentally affected by climate change^16^,^17^. It has been shown that changes in climate, especially temperature, can alter breeding phenology^15^,^18^,^19^,^20^,^21^,^22^, body condition^23^ and disease susceptibility^11^,^24^,^25^ of amphibians, but how and to what extent climatic changes may affect amphibians in hibernation has remained equivocal.

In the Northern Hemisphere temperate zone, climate change is projected to increase low-percentile (i.e. cold) winter temperatures and decrease the duration of cold periods and snow cover^16^,^17^. While amphibians are likely to benefit from shorter winters, the presumable effects of rising winter temperatures are less clear. It has been argued that milder winters could lead to amphibian declines by depleting the energy reserves of individuals due to a rise in metabolic rates and increased enzymatic activity, and negatively affecting survival and fecundity^14^,^23^. Other field studies, however, contradict the latter hypothesis by reporting higher mortality during winters with low and widely varying temperatures^26^,^27^,^28^. Nonetheless, separating climatic effects on population characteristics from other factors and identifying causal relationships remains a challenge for correlative studies, even if these span several years^24^,^29^. Experiments scrutinizing potential effects of climate change on amphibians have remained surprisingly scarce (for an exception see^30^). Further, studies on potential effects of climate change on amphibians generally neglect effects on juveniles (for exceptions see e.g.^27^,^30^), even though a sufficiently high survival rate and good body condition of juveniles are of fundamental importance for population persistence^27^,^31^,^32^.

Here we present a full factorial experimental test of the effects of rising winter temperature and shortening winter length on survival and body mass change of hibernating juveniles of an anuran amphibian of the temperate zone, the European common toad (*Bufo bufo*). We performed the experiment on a set of toadlets we had reared under controlled semi-natural conditions using mesocosms during the larval stage and outdoor enclosures after metamorphosis. In this way we controlled for individual variation in genetic relatedness and environmental conditions during early ontogeny that can have carry-over effects after metamorphosis^33^,^34^. We hibernated toadlets at current (1.5 °C) or elevated (4.5 °C) hibernation temperature in combination with current (91 days) or shortened (61 days) hibernation length. We weighed toadlets and documented mortality one week after termination of hibernation (Fig. 1).

## Results

### Survival during hibernation

Overall, 325 toadlets out of 371 (87.6%) survived hibernation (Fig. 2). Overwintering survival was higher in scenarios with the higher hibernation temperature and in scenarios with shorter hibernation period (Table 1, Fig. 2). Also, survival increased with larger body mass before hibernation (mean ± s.e.m. for survivors: 1389 ± 28.64 mg, N = 325; died: 1070 ± 52.57 mg, N = 46) and differed between larval environments (Table 1, Supplementary Table S1, Fig. S1). None of the tested interactions were significant (Supplementary Table S2). Number of toes clipped had no significant effect on survival (Supplementary Table S3).

### Body mass change during hibernation

Body mass one week after hibernation was positively related to mass before hibernation (linear mixed effects model, *t* = 36, *r* = 0.90, N = 325), and negatively to hibernation length (Table 1, Fig. 3). However, the interaction between hibernation temperature and hibernation length was also significant (Table 1): body mass of toadlets did not differ between cold and mild temperature regimes when exposed to shortened hibernation, but when toadlets were exposed to longer hibernation, animals overwintering at 4.5 °C weighed more after hibernation than toadlets that had overwintered at 1.5 °C (Fig. 3).

## Discussion

Survival of toadlets during overwintering was highest when exposed to a shortened hibernation period and elevated average winter temperature (61 days at 4.5 °C), and lowest under current average overwintering conditions (91 days at 1.5 °C). This result contradicts the hypothesis that milder winters could lead to increased mortality in poikilothermic amphibians due to elevated metabolic rates during a long period without feeding^14^,^23^. Positive effects of milder winter temperatures and/or shorter winters on overwintering survival of anurans have also been found by a few correlative studies^26^,^27^,^28^. The only other experimental study testing how climate change may affect hibernation success of an amphibian^30^ registered negligible mortality (3 out of 160 animals). Garner, *et al.*^30^ manipulated overwintering conditions by exposing juvenile common toads to different hibernation lengths of 51 and 11 days at 4 °C, which might have already exposed toads to more beneficial conditions than currently experienced in nature^35^,^36^,^37^, thereby possibly underestimating effects of climate change on hibernation success. Although we applied a fairly constant temperature treatment in our study, our results likely are valid for natural populations. Anholt, *et al.*^26^ found negative effects of highly variable winter temperatures on survival in a field study, but the recent report of the Intergovernmental Panel on Climate Change shows that low-percentile winter temperatures are predicted to increase faster than the mean, thereby decreasing temperature variability^17^. This way, if we had included variability in our treatments, our simulated climate change scenario would have been not only warmer but also less variable than the current climate scenario; so it is likely that we would have found even more pronounced differences in favor of the climate change group. Our results, thus, bolster up conclusions of previous correlative studies by providing experimental evidence for the hypothesis that temperate-zone anurans, including common toads, may benefit from mildly increasing temperatures due to climate change via increased winter survival. Nevertheless, further experiments are needed to scrutinize the effects of temperature variability coupled with warming.

Body mass after hibernation was very similar among toadlets hibernating for 61 days, regardless of temperature, and was higher than in animals hibernating for 91 days. This may have simply resulted from a shorter time period without food intake pertaining smaller loss of body mass. On the other hand, low temperatures seemed to enhance the negative effects of a long hibernation period. Glycogen and lipids are the main energy source for amphibians during hibernation^38^, but glycogen can also be utilized in the production of cryoprotective sugars^39^,^40^. Although there is no direct evidence that common toads can harness glycogen this way, they can survive subzero temperatures of −3 °C for up to 22 hours^40^. Thus, one possible contributor to the observed pattern may be that toadlets hibernating at 1.5 °C depleted their energy reserves twofold: by maintaining normal physiological functions and also deploying glycogen as a response to low temperature. Body mass before hibernation was also positively related to survival and post-hibernation mass, which is in accordance with results of a previous field-based study on juvenile anurans^33^. However, Garner, *et al.*^30^ did not find a significant effect of hibernation regime and mass before hibernation on proportional mass change of toadlets during hibernation, perhaps because overwintering conditions were benign in all of their scenarios (see above). Also, while in our study T_min_ was −0.77 °C, already low enough to trigger physiological mechanisms preventing freezing, Garner, *et al.*^30^ never exposed toads to temperatures approaching or below 0 °C (T_min_ was 4 °C), rendering the production of cryoprotective agents unnecessary.

In conclusion, under simulated current hibernation conditions in our study 67% of toadlets survived, which is within the range of overwintering survival estimated for natural conditions^26^,^28^,^41^, whereas under simulated climate change conditions survival was close to 100% one week after hibernation. Moreover, when exposed to a shorter and/or milder winter, survivors also had larger body mass after hibernation, which is likely to have long-lasting effects by enhancing further survival and fecundity^14^,^23^,^42^. While other consequences of climate change, such as an increasing probability of mass mortalities in aquatic larvae due to premature pond-desiccation, the spread of invasive predators and infectious diseases are likely to have negative effects on anuran populations located in the temperate zone and at high altitudes and latitudes^1^,^3^,^13^,^25^,^30^,^43^,^44^, the benefits arising from milder and shorter winters may somewhat dampen these effects on population persistence^27^,^31^,^32^. Further studies testing the general applicability of our results across several amphibian taxa, under different climate change scenarios and during several life stages are urgently needed. These investigations will deliver important insights regarding the expected effects of future climate change on amphibians, allowing the design and execution of informed and effective conservation actions protecting this highly threatened group of animals.

## Methods

### Ethics statement

Permits for collection and transport of animals were issued by the City of Vienna (MA22-120657/2014) and by Land Niederösterreich, Austria (RU5-BE-7/016-2014). Experimental procedures were approved by the institutional ethics committee and the national authority according to § 8ff of Law for Animal Experiments, Tierversuchsgesetz-TVG (GZ 68.205/0164-II/3b/2013) and were carried out in accordance with the approved guidelines.

### Experimental design

In late March 2014, we captured 12 amplexing common toad pairs and raised their offspring in full-sib groups of initially 60 individuals in 140 L outdoor plastic mesocosms until metamorphosis. Tadpoles developed in the presence or absence of a caged predator, represented by backswimmer imagos (*Notonecta* sp.), larvae of the southern hawker (*Aeshna cyanea*), juvenile sticklebacks (*Gasterosteus aculeatus*) and adult, male smooth newts (*Lissotriton vulgaris*).

We transferred metamorphosing toadlets into slightly tilted 45 L boxes containing egg boxes as shelter, and fed them *ad libitum* with springtails (*Folsomia* sp.), woodlice (*Trichorhina tomentosa*), fruit flies (*Drosophila hydei*) and hatchling crickets (*Gryllodes sigillatus*). After all individuals metamorphosed, we transported them to the Konrad Lorenz Institute of Ethology, Vienna, Austria. We marked toadlets of 10 families individually by toe-clipping, a method widely used for marking of anuran amphibians^45^,^46^, and assigned them randomly to 16 outdoor enclosures (set up similar to^33^). Each enclosure received one toadlet from each family by larval environment combination, resulting in 50 animals per enclosure.

**Figure 1:**
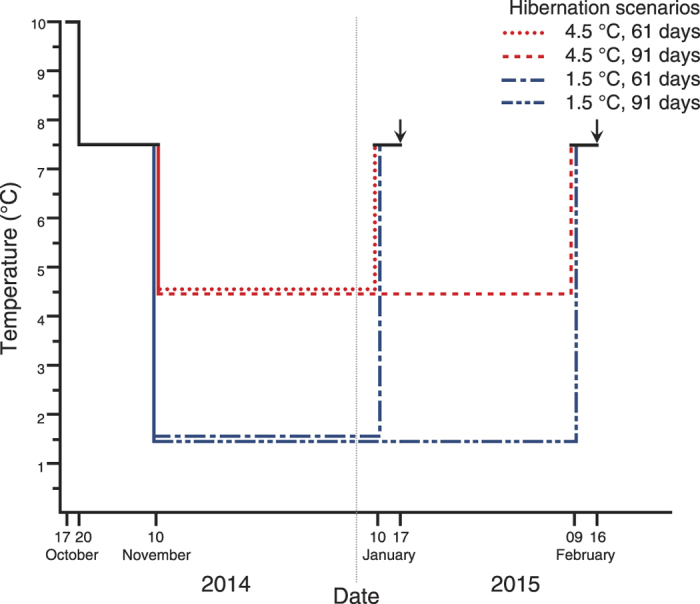
Schematic representation of the experimental procedures. Arrows denote the dates of weighing after hibernation. Temperature clines during the hibernation period are misaligned to allow visibility.

**Figure 2:**
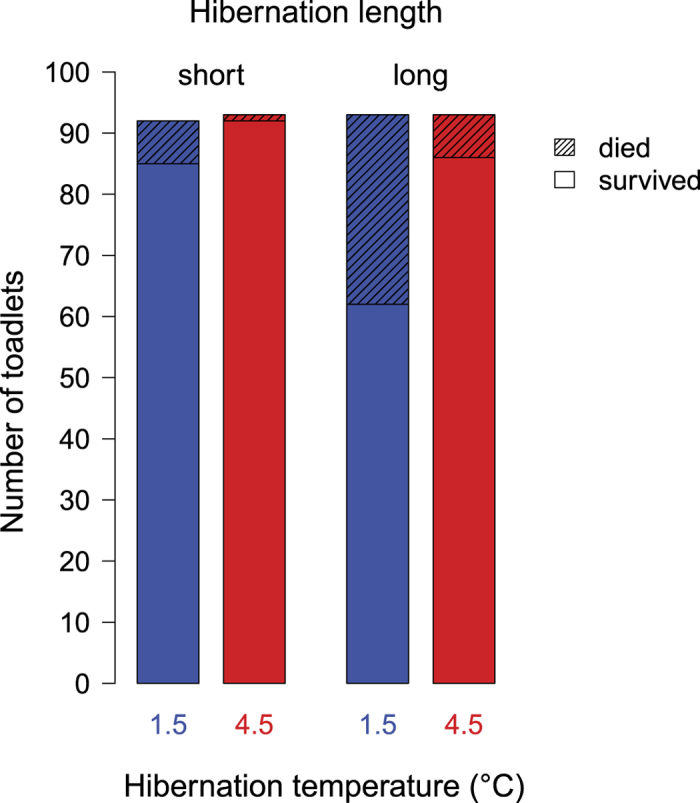
Number of toadlets that survived or died during hibernation.

**Figure 3:**
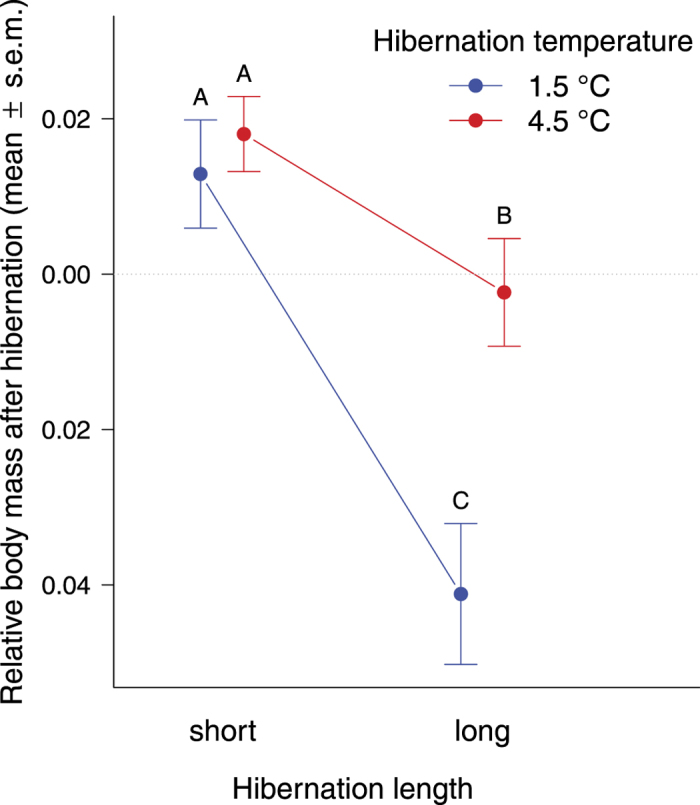
Body mass of toadlets relative to their pre-hibernation mass, one week after hibernation. The ‘Y’ axis represents residuals of a linear mixed-effects model consisting of body mass after hibernation as the dependent variable, body mass before hibernation as a covariate, and family crossed with enclosure as random factors. Mean ± s.e.m. values are shown (N = 325). Letters above error bars represent pairwise comparisons; groups marked with different letters differ significantly based on linear contrasts corrected for false discovery rate.

We recaptured toadlets from enclosures on 14–15 October and let them defecate in groups of 15–20 individuals in 45 L plastic outdoor containers filled with moist leaves to prevent desiccation. On 17 October after individual identification and measuring their body mass to the nearest mg, we transferred each toadlet separately into a 50 ml centrifuge tube (11.5 cm × 3 cm, length × diameter) filled with 20 ml of a 1:1 mixture of sterilized and dried (2 hours at 170 °C) soil and sand moistened with 5 ml aged tap water to allow burying and prevent desiccation. Tubes were covered with a mosquito net to allow air transfer and stored in a close to horizontal position (~5°). Toadlets had only a limited space available to move around during the experiment, but not much more may be available to animals in natural hibernacula^47^,^48^. We placed tubes into a lab refrigerator with forced air convection (Miele, Gütersloh, Germany) set to 10 °C. Three days later, we lowered the temperature to 7.5 °C and maintained it for three weeks to simulate transient autumn temperatures (Fig. 1).

We randomly selected 372 individuals out of 403 survivors and assigned 93 toadlets randomly to each of the four hibernation scenarios, while balancing for body mass, larval environment, family and enclosure (for details see Supplementary Table S4). The simulated current average hibernation temperature of 1.5 °C is well within the natural range experienced by common toads during winter^35^,^41^ and a 3 °C increase in hibernation temperature (from the current 1.5 °C to 4.5 °C) could be expected to be of significance for the toadlets’ performance during hibernation^36^,^37^, while not exceeding projected temperature rise due to climate change^17^ (for further justification of the chosen temperatures see the Supplementary Information). We set the length of the current hibernation scenario based on average monthly temperatures registered in Hungary between November and March during the period of 1950–2000^49^, and on reported durations of hibernation in common toads^35^,^41^. Shortening of the hibernation period by 30 days is supported by previous publications on shorter snow-cover duration^50^ and elongated growing season in the Northern Hemisphere^51^.

We initiated hibernation on 10 November 2015 by relocating toadlets into two laboratory refrigerators with forced air convection (Pol-Eko-Aparatura, Wodzisław Śląski, Poland). We recorded temperature hourly using data loggers (Onset, Cape Cod, MA, USA; for measured temperatures see Supplementary Table S5). Once a week we sprinkled aged tap water into tubes to prevent desiccation and relocated tubes within refrigerators haphazardly to account for spatial variation in temperature. Sixty-one and 91 days after initiation of hibernation, we transferred toadlets from both 1.5 and 4.5 °C to a refrigerator set to 7.5 °C (Fig. 1). Apart from phases of sprinkling and relocating, toadlets overwintered in complete darkness and without food throughout the study. For further details on the materials and methodology see the online Supplementary Information and Supplementary Table S4

### Statistical analyses

We analyzed survival using generalized linear mixed-effects modeling procedures with binomial error distribution and logit link function. Effects on post-hibernation body mass were evaluated using general linear mixed-effects models. Mass was log_10_-transformed prior to analysis. We applied a forward model-selection approach based on *P-*values (α = 0.05), testing the main effects of larval environment, hibernation length and hibernation temperature as fixed factors, body mass before hibernation as a covariate, tadpole family and enclosure as crossed random factors, and all testable two- and three-way interactions among fixed effects, including the covariate. We used a forward model-selection approach to ensure a standardized method of analysis for all studied response variables, since some interactions with larval environment could not be estimated for survival because in some subgroups there was no variance (all individuals survived or died, Supplementary Tables S2, S6, S7), which rendered backwards model selection unfeasible. We included larval environment as a fixed effect in the analysis to control for potential carry-over effects after metamorphosis^33^,^34^. We ran all analyses in R 3.1.3^52^. For the analysis of survival, we used the ‘glmer’ function in the ‘lme4’ package^53^, with the ‘bobyqa’ optimizer function to handle computational problems arising during the model-selection process. When analyzing variation in body mass we used the ‘lmer’ function in ‘lme4’. *P*-values were calculated using ‘Anova’ in the ‘car’ package^54^. We conducted post-hoc pairwise comparisons by calculating linear contrasts corrected for false discovery rate^55^ using ‘glht’ in the ‘multcomp’ package^56^. We discarded one toadlet from the analyses, due to missing data (Supplementary Table S4).

## Additional Information

**How to cite this article**: Üveges, B. *et al.* Experimental evidence for beneficial effects of projected climate change on hibernating amphibians. *Sci. Rep.* **6**, 26754; doi: 10.1038/srep26754 (2016).

## Supplementary Material

Supplementary Information

## Figures and Tables

**Table 1:** Significant (*P* < 0.05) main effects and interaction affecting survival and body mass change of juvenile common toads (*B. bufo*) during hibernation.

	n	*χ*^*2*^	df	*P*
Survival during hibernation				
intercept	371	17.52	1	<0.001
hibernation length		23.60	1	<0.001
hibernation temperature		24.53	1	<0.001
larval environment		10.67	4	0.031
mass before hibernation		21.32	1	<0.001
Body mass after hibernation				
intercept	325	0.71	1	0.401
hibernation length		30.99	1	<0.001
hibernation temperature		0.36	1	0.549
mass before hibernation		1296.06	1	<0.001
hibernation length × hibernation temperature		6.38	1	0.012

Results were obtained from type III analysis-of-deviance tables with Wald χ^2^ tests. Non-significant terms can be found in Supplementary Table S2.
