# Supplementary material for: Experimental evidence for beneficial effects of projected climate change on hibernating amphibians
Source: Sci Rep. 2016 May 27;6:26754. doi: 10.1038/srep26754 (PMC4882615; doi:10.1038/srep26754)

Supplementary Information for

Experimental evidence for beneficial effects of projected climate change on hibernating amphibians

Bálint Üveges, Katharina Mahr, Márk Szederkényi, Veronika Bókony, Herbert Hoi, Attila Hettyey

Table of contents

Supplementary Methods **3**
Detailed experimental design 3
Rationale for selected hibernation temperatures 4
Supplementary Results **5**
Survival and body mass change after hibernation 5
Effects of the larval environment 6
Supplementary Discussion **6**
Supplementary References **7**
Supplementary Tables **9**
Table S1 9
Table S2 10
Table S3 11
Table S4 12
Table S5 26
Table S6 27
Table S7 28
Supplementary Figures **29**
Figure S1 29
Figure S2 30
Figure S3 31
Figure S4 32
Figure S5 33

Supplementary Methods

Detailed experimental design

We captured common toad pairs at Silbersee, Vienna, Austria (48°12’33”N, 16°15’48”E) and transported them to the premises of the PNMS/PHS Sacré Coeur in Pressbaum, Austria (48°11’11”N, 16°04’48”E) where we allowed pairs to lay eggs in 45 L plastic boxes and raised their offspring until metamorphosis in 140 L mesocosms (82 × 58 × 30 cm, length × width × height). Mesocosms were set up two weeks before the addition of toad eggs by filling with 130 L of aged tap-water, adding 50 g dried beech leaves, and inoculating with 1 L of pond water (for similar methodology see e.g. [1,2]). The resulting density of 60 tadpoles per 0.13 m^3 water (~460 tadpoles/m^3) falls well within the range that can be observed in natural ponds in the area (Bókony et al. unpubl. data).

We collected backswimmer imagos (*Notonecta* sp.), 4th instar larvae of the southern hawker (*Aeshna cyanea*) and adult, male smooth newts (*Lissotriton vulgaris*) from private ponds in Lower-Austria, Austria, and we commercially bought juvenile three-spined sticklebacks (*Gasterosteus aculeatus*). In the experiment we fed caged specimens of each predator with one *B. bufo* and one *Rana temporaria* tadpole three times a week. All four species represent naturally occurring predators of *B. bufo*.

After metamorphosis, toadlets were kept at the Konrad Lorenz Institute of Ethology (KLIVV), Vienna, Austria (48°13’16”N, 16°17’06”E). We excluded two families from further stages of the study, because of low numbers of surviving toadlets. Enclosures were located in a forested area of the KLIVV. They measured 3 × 3 m, were 60 cm high and buried 20 cm into the ground. The walls were made of 80 cm wide mosquito screen fixed on a wooden frame, with a 10 cm wide mosquito net strip fixed on top. This allowed prey items and aerial predators entering from above, but kept toadlets inside and terrestrial competitors and predators outside. Enclosures contained two shallow plastic dishes filled with water and

varying density of natural vegetation. We supplemented naturally arriving prey by adding a mixture of the arthropods also used as food immediately after metamorphosis (see in the main text) and later also mealworms (larval *Tenebrio molitor*) two times a week. Due to fissures on the wall of three enclosures and grass snakes (*Natrix natrix*) entering a fourth enclosure, recapture rates at the end of the growing season turned out to be very low in four enclosures (8-34 %), leaving us with toadlets from 12 enclosures to be used in the subsequent hibernation experiment.

We minimized handling of toadlets throughout hibernation to maintain stable temperatures in the refrigerators and to reduce handling stress, thus the tubes of toads were only moistened and relocated during hibernation, but no data regarding survival or body mass was taken at these regular occasions. Our goal in this study was to estimate survival until the end of hibernation, not to analyze the dynamics of survival during this period, since the number of surviving toadlets that can continue their lives after hibernation is the relevant factor for the long-term persistence of populations. Similarly, a second sampling occasion (3 weeks after the end of hibernation) was introduced to enable the estimation of recovery after overwintering and delayed effects of hibernation conditions, without studying mortality patterns between the two sampling occasions.

After the termination of the experiment we kept toadlets at 12 °C in large boxes, and fed them with commercially bought juvenile crickets and woodlice *ad libitum* until the weather permitted their release at their pond of origin in mid March.

Rationale for selected hibernation temperatures

In a field study Sinsch [3] registered ambient temperatures between 0.9 and 3.1 °C within hibernation sites of surviving animals, while van Gelder, et al. [4] found that minimum body temperatures of common toads ranged between 0.6 and 3.3 °C. Gittins, et al. [5] and Denton and

Beebee [6] observed a threshold of movement and activity of toads at 4 °C. At the same time, climate change projections predict an increase in winter temperatures by 1-4 °C for Central and Eastern Europe by the end of the century [7].

Supplementary Results

Survival and body mass change after hibernation

After the post-hibernation identification and weighing of toadlets we kept them in the refrigerator set to 7.5 °C for another two weeks and documented mortality and weighed them again at the end of this period. We calculated body mass change between the two post-hibernation weighing occasion as $\log_{10}$(mass three weeks after hibernation/mass one week after hibernation). Statistical analysis of these data commenced identically as presented in the *Materials and Methods* section of the main text. Since results obtained from the data collected in this period of the study do not contradict results and conclusions presented in the main text, we mention them briefly only here.

Between the two post-hibernation weighing occasions 28 toadlets died (Fig. S2). During our analysis we found that survival in this period was affected by the three-way interaction of hibernation temperature, hibernation length and body mass before hibernation (Table S6). However, this interaction was due to three toadlets in the 4.5 °C, short hibernation scenario that had very large pre-hibernation mass, experienced large mass loss during hibernation, and died after the first weighing (no further toadlets died after hibernation in this scenario; Fig. S3). To ensure that our conclusions are not biased by a few outliers, we reduced this model by excluding the three-way interaction and both two-way interactions of mass before hibernation (with temperature during hibernation and hibernation length, respectively). After reducing the model, we found that survival in this period was higher when winter was milder (Table S7, Fig. S2), and tended to be higher when winter was shorter (Wald $\chi^2_1 = 2.76$,

P = 0.097), whereas the interaction between hibernation temperature and hibernation length was not significant (Wald χ^2_1 = 0.29, P = 0.592) (Table S7, Fig. S2).

Body mass change after hibernation in the remaining 297 specimens was significantly affected by hibernation length, hibernation temperature and their interaction (Table S7). Toadlets experiencing a shorter hibernation period gained mass after hibernation, whereas those overwintering for longer lost mass (Fig. S4); mass gain was larger after a short mild winter than after a short cold winter while there was no effect of hibernation temperature when the winter was long (Fig. S4).

Effects of the larval environment

Individuals that had developed in the presence of *A. cyanea* as tadpoles had a higher survival rate during hibernation than control tadpoles that had developed in the absence of predators (95.1 % and 83.6 % survived, respectively; Table S1 and Fig. S1). We also found a marginally non-significant effect of larval environment (Wald χ^2_1 = 9.36, P = 0.053) on body mass change after hibernation, where toads that had developed in the presence of *A. cyanea* as tadpoles showed higher mass gain compared to controls (mass change as means ± S.E., toads reared with *A. cyanea*: 128 ± 27.15 mg, N = 68; controls: 70 ± 25.53 mg, N = 57; Table S7 and Fig. S5).

Supplementary Discussion

Body mass gain of toadlets between the two weighing occasions after hibernation could have resulted from physiological changes as an adjustment to spring conditions, such as restoration of the water balance by water uptake from the environment. At the same time, lower temperatures may have impeded physiological reactions to the onset of spring in toadlets hibernating at 1.5 °C for 61 days compared to toadlets overwintering at 4.5 °C for the same

length, resulting in less mass gained. Short bouts of freezing temperatures are known to prolong recovery and seriously reduce performance of anurans after hibernation [8,9]. It is possible that long exposure to relatively low temperatures, as in our study, acts similarly, but this hypothesis remains to be tested.

Presence of *A. cyanea* larvae during the tadpole stage enhanced survival of toadlets during hibernation, and tended to increase post-hibernation mass gain compared to control individuals. This supports the conclusion of Garner, et al. [10], that survival during winter can be affected by larval traits. Altwegg and Reyer [11] and Relyea [12] also reported that timing of, and size at metamorphosis, which are known to be influenced by cues on predation threat, can have long lasting effects on morphology and survival of anuran amphibians.

Supplementary References

1 Hettyey, A., Vincze, K., Zsarnóczai, S., Hoi, H. & Laurila, A. Costs and benefits of defences induced by predators differing in dangerousness. *J. Evol. Biol.* **24,** 1007-1019 (2011).

2 Van Buskirk, J. Incipient habitat race formation in an amphibian. *J. Evol. Biol.* **27,** 585-592 (2014).

3 Sinsch, U. Seasonal changes in the migratory behaviour of the toad *Bufo bufo*: direction and magnitude of movements. *Oecologia* **76,** 390-398 (1988).

4 van Gelder, J. J., Olders, J. H. J., Bosch, J. W. G. & Starmans, P. W. Behaviour and body temperature of hibernating common toads *Bufo bufo*. *Holarctic Ecol.* **9,** 225-228 (1986).

5 Gittins, S. P., Parker, A. G. & Slater, F. M. Population characteristics of the common toad (*Bufo bufo*) visiting an breeding site in mid-Wales. *J. Anim. Ecol.* **49,** 161-173 (1980).

6 Denton, J. S. & Beebee, T. J. C. Summer and winter refugia of natterjacks (*Bufo calamita*) and common toads (*Bufo bufo*) in Britain. *Herpetol. J.* **3,** 90-94 (1993).

7 IPCC. *Climate Change 2013: The Physical Science Basis. Contribution of Working Group I to the Fifth Assessment Report of the Intergovernmental Panel on Climate*

Change. (Cambridge University Press, Cambridge, United Kingdom and New York, NY, USA, 2013).

8 Costanzo, J., P., Irwin, J., T. & Lee, R. E., Jr. Freezing impairment of male reproductive behaviors of the freeze-tolerant wood frog, *Rana sylvatica*. *Physiol. Zool.* **70,** 158-166 (1997).

9 Voituron, Y. & Lengagne, T. Freezing before mating severely affects the fitness of a capital breeder. *Physiol. Biochem. Zool.* **81,** 204-210 (2008).

10 Garner, T. W. J., Rowcliffe, J. M. & Fisher, M. C. Climate change, chytridiomycosis or condition: an experimental test of amphibian survival. *Glob. Change Biol.* **17,** 667-675 (2011).

11 Altwegg, R. & Reyer, H.-U. Patterns of natural selection on size at metamorphosis in water frogs. *Evolution* **57,** 872-882 (2003).

12 Relyea, R. A. The lasting effects of adaptive plasticity: predator-induced tadpoles become long-legged frogs. *Ecology* **82,** 1947-1955 (2001).

Table S1. Effects of larval environment (in bold) on the survival of toadlets during hibernation (N=371). Generalized linear mixed-effects model fit by maximum likelihood (Laplace Approximation) using the 'summary' function in the 'lme4' package in R. Intercept refers to control toadlets (reared without predators as tadpoles) hibernating at 1.5 °C for 61 days.

	Estimate	s.e.m.	*z*	*P*
intercept	-21.2290	5.0726	-4.185	<0.001
hibernation temperature (4.5 °C)	2.6130	0.5276	4.952	<0.001
hibernation length (91 days)	-2.4364	0.5016	-4.857	<0.001
***Notonecta* sp.**	0.9320	0.6708	1.389	0.165
Aeshna cyanea	1.8990	0.7383	2.572	0.01
Gasterosteus aculeatus	-0.2674	0.5662	-0.472	0.637
Lissotriton vulgaris	0.4597	0.5836	0.788	0.431
mass before hibernation	7.7514	1.6789	4.617	<0.001

Table S2. Non-significant main effects and interactions in analyses including survival and body mass after hibernation. Each effect was calculated by including that effect into the final models shown in Table 1. Results were obtained from type III analysis-of-deviance tables with Wald χ^2 tests using the car package in R. In case of survival, interactions with larval environment could not be estimated because in some subgroups there was no variance (all individuals survived or died), but corresponding graphs did not suggest the presence of significant interactions between larval environment and the other explanatory variables. Graphs are available from the authors upon request.

	N	χ^2	Df	*P*
Survival during hibernation	371			
hibernation length × hibernation temperature		0.03	1	0.868
hibernation length × mass before hibernation		0.43	1	0.514
hibernation temperature × mass before hibernation		0.61	1	0.436
Body mass after hibernation	325			
larval environment		2.48	4	0.648
hibernation length × larval environment		5.19	4	0.268
hibernation length × mass before hibernation		2.11	1	0.147
hibernation temperature × larval environment		4.05	4	0.399
hibernation temperature × mass before hibernation		0.59	1	0.441
larval environment × mass before hibernation		3.28	4	0.512
hibernation length × hibernation temperature × mass before hibernation		2.67	1	0.102
hibernation length × larval environment × mass before hibernation		4.98	4	0.289
hibernation temperature × larval environment × mass before hibernation		2.03	4	0.730
larval environment × hibernation length × hibernation temperature		0.85	4	0.932

Table S3. Effect of toe clipping on survival of toadlets during and after hibernation. Number of toes clipped had no significant effect on survival either during (Wald $\chi^2_2 = 2.09$, $P = 0.352$) or after hibernation (Wald $\chi^2_2 = 4.58$, $P = 0.101$).

Number of toes clipped	During hibernation			After hibernation		
	N	Survivors	% survived	N	Survivors	% survived
1	76	65	85.53	65	61	93.85
2	270	236	87.41	236	217	91.95
3	25	24	96	24	19	79.17

Table S4. Family, larval environment, enclosure, and mass before hibernation of common toad (*Bufo bufo*) toadlets used in the hibernation experiment. Specimen 378 was discarded from the analysis, due to missing data.

ID number	Family	Larval environment	Enclosure	Mass before hibernation (mg)	Hibernation length	Hibernation temperature	Survived 1 week after hibernation	Survived 3 weeks after hibernation	1st mass after hibernation	2nd mass after hibernation
1	8	*Aeshna cyanea*	1	1551	61 days	1.5 °C	Yes	Yes	1480	1665
2	8	*Lissotriton vulgaris*	1	1359	91 days	4.5 °C	Yes	Yes	1365	1338
3	3	*Notonecta* sp.	1	1391	61 days	4.5 °C	Yes	Yes	1273	1402
4	9	*Notonecta* sp.	1	2693	61 days	1.5 °C	Yes	Yes	1897	2280
5	2	*Aeshna cyanea*	1	1363	91 days	4.5 °C	Yes	Yes	1329	1590
6	4	*Aeshna cyanea*	1	1126	61 days	4.5 °C	Yes	Yes	978	1073
7	5	*Gasterosteus aculeatus*	1	1479	91 days	4.5 °C	Yes	Yes	1030	948
8	1	*Gasterosteus aculeatus*	1	2234	61 days	1.5 °C	Yes	Yes	2140	2178
9	3	*Aeshna cyanea*	1	1303	91 days	1.5 °C	Yes	Yes	1241	1322
10	10	*Aeshna cyanea*	1	2436	91 days	1.5 °C	Yes	Yes	1496	1635
11	9	*Gasterosteus aculeatus*	1	2124	61 days	4.5 °C	Yes	Yes	2117	2376
12	1	control	1	1893	91 days	4.5 °C	Yes	Yes	1656	1346
13	8	control	1	1226	91 days	4.5 °C	Yes	Yes	1312	762
14	8	*Gasterosteus aculeatus*	1	1160	61 days	4.5 °C	Yes	Yes	869	917
15	10	*Gasterosteus aculeatus*	1	2051	61 days	1.5 °C	Yes	Yes	1554	1939
16	5	*Aeshna cyanea*	1	1390	61 days	1.5 °C	Yes	No	782	NA
17	7	*Aeshna cyanea*	1	1226	91 days	4.5 °C	Yes	Yes	1143	1084
18	1	*Lissotriton vulgaris*	1	1793	61 days	1.5 °C	Yes	Yes	1387	1625
19	3	*Gasterosteus aculeatus*	1	1396	91 days	1.5 °C	Yes	Yes	1070	889
20	6	*Lissotriton vulgaris*	1	2052	61 days	4.5 °C	Yes	No	1325	NA
21	4	*Lissotriton vulgaris*	1	1394	61 days	4.5 °C	Yes	Yes	1054	1247

22	9	*Lissotriton vulgaris*	1	1728	91 days	4.5 °C	Yes	No	1245	NA
23	3	*Lissotriton vulgaris*	1	1227	61 days	4.5 °C	Yes	Yes	1064	1139
24	10	*Lissotriton vulgaris*	1	1795	61 days	1.5 °C	Yes	Yes	1658	1674
25	9	*Aeshna cyanea*	1	2215	61 days	1.5 °C	Yes	Yes	1425	1294
26	3	control	1	1420	91 days	4.5 °C	Yes	No	1243	NA
27	2	*Lissotriton vulgaris*	1	1395	91 days	4.5 °C	Yes	Yes	1105	836
28	7	*Gasterosteus aculeatus*	1	1555	61 days	4.5 °C	Yes	Yes	1473	1467
29	10	control	1	2582	91 days	1.5 °C	Yes	Yes	1821	1763
30	5	*Notonecta* sp.	1	1112	61 days	4.5 °C	Yes	Yes	918	895
31	5	*Lissotriton vulgaris*	1	1398	61 days	4.5 °C	Yes	Yes	1117	1340
32	7	*Lissotriton vulgaris*	1	1156	61 days	1.5 °C	Yes	Yes	1191	1138
33	10	*Notonecta* sp.	1	3200	61 days	4.5 °C	Yes	Yes	2580	2771
34	7	control	1	1462	91 days	1.5 °C	No	NA	NA	NA
35	5	*Aeshna cyanea*	2	2295	61 days	4.5 °C	Yes	Yes	1903	2574
36	10	*Notonecta* sp.	2	4245	91 days	4.5 °C	Yes	Yes	3917	3608
37	1	*Aeshna cyanea*	2	1793	91 days	1.5 °C	Yes	Yes	1498	1458
38	9	*Gasterosteus aculeatus*	2	2793	91 days	4.5 °C	Yes	Yes	2469	2494
39	4	*Lissotriton vulgaris*	2	1196	91 days	4.5 °C	Yes	Yes	1007	1033
40	7	control	2	1634	91 days	4.5 °C	Yes	Yes	1252	1083
41	8	*Gasterosteus aculeatus*	2	3470	91 days	1.5 °C	Yes	Yes	2597	2550
42	4	*Aeshna cyanea*	2	1813	61 days	4.5 °C	Yes	Yes	1656	1775
43	6	*Lissotriton vulgaris*	2	1688	91 days	4.5 °C	Yes	Yes	1318	1259
44	8	*Lissotriton vulgaris*	2	2168	91 days	4.5 °C	Yes	Yes	1668	1474
45	9	*Aeshna cyanea*	2	1776	91 days	1.5 °C	Yes	Yes	1893	1888
46	6	*Notonecta* sp.	2	1956	61 days	4.5 °C	Yes	No	1115	NA
47	7	*Lissotriton vulgaris*	2	2198	61 days	1.5 °C	Yes	No	1280	NA
48	2	control	2	2041	61 days	1.5 °C	Yes	Yes	1736	1925
49	3	control	2	1334	61 days	1.5 °C	Yes	Yes	1027	1170

50	4	*Gasterosteus aculeatus*	2	1504	61 days	1.5 °C	Yes	Yes	962	1187
52	9	*Notonecta* sp.	2	2009	61 days	4.5 °C	Yes	Yes	1899	1777
53	10	*Aeshna cyanea*	2	2557	91 days	4.5 °C	Yes	Yes	2266	2018
54	6	*Gasterosteus aculeatus*	2	1485	61 days	1.5 °C	Yes	Yes	1267	1363
55	6	control	2	1183	61 days	1.5 °C	Yes	Yes	1049	1171
56	3	*Gasterosteus aculeatus*	2	1555	91 days	1.5 °C	No	NA	NA	NA
57	4	control	2	2418	61 days	1.5 °C	Yes	Yes	2228	2283
58	10	*Gasterosteus aculeatus*	2	3221	61 days	4.5 °C	Yes	Yes	2484	2447
59	5	control	2	1481	91 days	1.5 °C	No	NA	NA	NA
71	5	*Lissotriton vulgaris*	3	1255	61 days	1.5 °C	Yes	Yes	1122	1227
72	5	*Gasterosteus aculeatus*	3	1505	61 days	4.5 °C	Yes	Yes	1384	1356
73	4	*Lissotriton vulgaris*	3	1211	61 days	1.5 °C	Yes	Yes	1263	1288
74	9	*Lissotriton vulgaris*	3	1509	91 days	4.5 °C	Yes	Yes	1474	1573
75	1	control	3	1660	61 days	4.5 °C	Yes	Yes	1719	1750
76	6	*Aeshna cyanea*	3	1341	61 days	1.5 °C	Yes	Yes	1221	1233
77	6	*Notonecta* sp.	3	1329	61 days	1.5 °C	Yes	Yes	1144	1363
78	3	*Aeshna cyanea*	3	971	61 days	1.5 °C	Yes	Yes	824	1041
79	2	*Gasterosteus aculeatus*	3	1576	91 days	1.5 °C	Yes	Yes	1256	1251
80	8	*Notonecta* sp.	3	1123	91 days	1.5 °C	Yes	Yes	778	703
81	1	*Notonecta* sp.	3	1513	61 days	4.5 °C	Yes	Yes	1308	1278
82	10	*Gasterosteus aculeatus*	3	2214	91 days	1.5 °C	Yes	Yes	1580	1448
83	4	*Gasterosteus aculeatus*	3	1282	91 days	4.5 °C	Yes	Yes	1306	1141
84	5	*Notonecta* sp.	3	1345	91 days	4.5 °C	Yes	Yes	1152	1031
85	2	*Notonecta* sp.	3	1243	91 days	1.5 °C	Yes	Yes	1083	1262
86	10	control	3	1255	91 days	4.5 °C	No	NA	NA	NA
87	2	*Aeshna cyanea*	3	1433	61 days	4.5 °C	Yes	Yes	1168	1319
88	3	*Gasterosteus aculeatus*	3	1220	61 days	4.5 °C	Yes	Yes	1100	1339
89	1	*Aeshna cyanea*	3	1545	91 days	4.5 °C	Yes	Yes	1300	1366

90	2	*Lissotriton vulgaris*	3	1508	91 days	1.5 °C	Yes	Yes	1177	1201
91	6	*Lissotriton vulgaris*	3	1218	91 days	1.5 °C	No	NA	NA	NA
92	9	*Notonecta* sp.	3	2174	91 days	4.5 °C	Yes	Yes	1858	1469
93	4	*Aeshna cyanea*	3	1543	91 days	1.5 °C	Yes	No	888	NA
94	7	*Gasterosteus aculeatus*	3	1917	61 days	1.5 °C	Yes	Yes	1803	1842
95	4	control	3	1004	91 days	1.5 °C	Yes	No	653	NA
96	9	*Gasterosteus aculeatus*	3	2478	91 days	4.5 °C	Yes	Yes	2331	2255
97	10	*Notonecta* sp.	3	902	91 days	4.5 °C	Yes	Yes	830	811
98	8	*Gasterosteus aculeatus*	3	1099	91 days	1.5 °C	No	NA	NA	NA
99	6	control	3	1240	91 days	1.5 °C	No	NA	NA	NA
100	4	*Notonecta* sp.	3	1265	91 days	4.5 °C	Yes	Yes	1319	1339
101	10	*Lissotriton vulgaris*	3	1825	91 days	1.5 °C	Yes	Yes	1437	1246
102	2	control	3	1264	91 days	1.5 °C	Yes	Yes	1072	1053
103	7	*Aeshna cyanea*	3	1364	61 days	4.5 °C	Yes	Yes	1035	1371
104	8	*Aeshna cyanea*	3	1002	61 days	1.5 °C	Yes	Yes	842	925
105	3	control	3	796	61 days	4.5 °C	Yes	Yes	731	865
106	5	*Aeshna cyanea*	3	1126	61 days	4.5 °C	Yes	Yes	1062	1121
107	10	*Notonecta* sp.	4	3089	61 days	1.5 °C	Yes	Yes	2515	2465
108	1	*Gasterosteus aculeatus*	4	1909	91 days	4.5 °C	Yes	Yes	1782	1698
109	1	control	4	1146	61 days	4.5 °C	Yes	Yes	1167	1341
110	4	*Notonecta* sp.	4	1117	61 days	1.5 °C	Yes	Yes	1045	1083
111	3	*Aeshna cyanea*	4	959	91 days	1.5 °C	No	NA	NA	NA
112	8	*Notonecta* sp.	4	1573	91 days	4.5 °C	Yes	Yes	1217	1079
113	2	*Gasterosteus aculeatus*	4	2130	61 days	1.5 °C	Yes	Yes	2067	2109
114	3	*Gasterosteus aculeatus*	4	1112	91 days	4.5 °C	Yes	Yes	754	666
115	9	control	4	2950	91 days	1.5 °C	Yes	Yes	2042	1942
116	2	control	4	948	91 days	4.5 °C	Yes	Yes	669	553
117	9	*Aeshna cyanea*	4	1097	61 days	4.5 °C	Yes	Yes	1006	1310

118	5	*Aeshna cyanea*	4	1142	91 days	1.5 °C	Yes	Yes	956	1071
119	4	*Aeshna cyanea*	4	831	61 days	4.5 °C	Yes	Yes	839	933
120	8	*Gasterosteus aculeatus*	4	1399	91 days	4.5 °C	Yes	Yes	1127	1200
121	7	*Gasterosteus aculeatus*	4	1193	91 days	1.5 °C	No	NA	NA	NA
122	5	control	4	870	91 days	1.5 °C	No	NA	NA	NA
123	4	control	4	1086	91 days	4.5 °C	Yes	Yes	808	775
124	10	*Aeshna cyanea*	4	1230	61 days	1.5 °C	Yes	Yes	1151	1365
125	2	*Aeshna cyanea*	4	1095	91 days	4.5 °C	Yes	Yes	1121	1188
126	7	*Lissotriton vulgaris*	4	1131	91 days	1.5 °C	Yes	Yes	708	670
127	5	*Notonecta* sp.	4	927	61 days	1.5 °C	Yes	Yes	918	942
128	8	*Aeshna cyanea*	4	980	91 days	4.5 °C	Yes	Yes	872	901
129	7	*Aeshna cyanea*	4	1046	61 days	1.5 °C	Yes	Yes	957	1061
130	5	*Lissotriton vulgaris*	4	1167	61 days	4.5 °C	Yes	Yes	1017	1400
131	6	*Gasterosteus aculeatus*	4	1340	91 days	4.5 °C	Yes	Yes	1091	1079
132	9	*Notonecta* sp.	4	1253	91 days	1.5 °C	Yes	Yes	1052	1121
133	2	*Notonecta* sp.	4	1251	91 days	4.5 °C	Yes	Yes	1126	1264
134	1	*Aeshna cyanea*	4	1456	91 days	1.5 °C	Yes	Yes	1122	1042
135	6	*Notonecta* sp.	4	1363	61 days	4.5 °C	Yes	Yes	1230	1463
137	6	*Lissotriton vulgaris*	4	1218	91 days	1.5 °C	Yes	Yes	939	1476
138	5	*Aeshna cyanea*	5	1482	91 days	4.5 °C	Yes	Yes	862	1200
139	8	*Gasterosteus aculeatus*	5	1196	61 days	1.5 °C	Yes	Yes	1117	1278
140	10	control	5	1529	61 days	1.5 °C	Yes	Yes	1229	1489
141	4	control	5	1405	61 days	4.5 °C	Yes	Yes	1155	1577
142	9	*Gasterosteus aculeatus*	5	1715	91 days	1.5 °C	No	NA	NA	NA
143	7	control	5	2089	61 days	1.5 °C	Yes	Yes	1867	2110
144	3	*Gasterosteus aculeatus*	5	1691	61 days	1.5 °C	Yes	Yes	1584	1714
145	2	*Notonecta* sp.	5	1419	91 days	1.5 °C	Yes	Yes	974	1079
146	10	*Lissotriton vulgaris*	5	1310	91 days	1.5 °C	No	NA	NA	NA

147	2	*Gasterosteus aculeatus*	5	1745	61 days	4.5 °C	Yes	Yes	1724	1864
148	1	*Notonecta* sp.	5	1389	91 days	1.5 °C	Yes	Yes	1091	1175
149	2	*Aeshna cyanea*	5	1444	91 days	1.5 °C	Yes	Yes	1278	1301
150	10	*Gasterosteus aculeatus*	5	1432	61 days	4.5 °C	Yes	Yes	1136	1660
151	1	*Gasterosteus aculeatus*	5	1331	91 days	1.5 °C	Yes	Yes	832	870
152	9	*Lissotriton vulgaris*	5	1689	91 days	1.5 °C	Yes	Yes	984	944
153	4	*Aeshna cyanea*	5	1884	91 days	1.5 °C	Yes	No	1095	NA
154	10	*Aeshna cyanea*	5	1864	61 days	4.5 °C	Yes	Yes	1662	2526
155	2	control	5	1620	61 days	4.5 °C	Yes	Yes	1479	1815
156	6	*Gasterosteus aculeatus*	5	1403	91 days	1.5 °C	No	NA	NA	NA
157	8	*Aeshna cyanea*	5	1396	91 days	4.5 °C	Yes	Yes	1441	1219
158	6	control	6	795	91 days	4.5 °C	No	NA	NA	NA
159	5	*Notonecta* sp.	6	1022	61 days	4.5 °C	Yes	Yes	865	1107
160	5	*Lissotriton vulgaris*	6	813	61 days	4.5 °C	Yes	Yes	668	1031
161	2	control	6	628	61 days	4.5 °C	Yes	Yes	567	693
162	10	*Lissotriton vulgaris*	6	1771	91 days	1.5 °C	No	NA	NA	NA
163	7	*Gasterosteus aculeatus*	6	967	91 days	1.5 °C	No	NA	NA	NA
164	3	*Gasterosteus aculeatus*	6	1299	61 days	1.5 °C	No	NA	NA	NA
165	9	*Notonecta* sp.	6	1055	61 days	1.5 °C	No	NA	NA	NA
166	10	*Gasterosteus aculeatus*	6	1228	61 days	4.5 °C	Yes	Yes	962	1098
167	10	*Aeshna cyanea*	6	1532	61 days	4.5 °C	Yes	Yes	1012	1240
168	7	*Aeshna cyanea*	6	1133	61 days	4.5 °C	Yes	Yes	1051	1208
169	4	control	6	2208	91 days	4.5 °C	Yes	Yes	1847	1656
170	6	*Notonecta* sp.	6	1277	91 days	1.5 °C	Yes	Yes	984	724
171	7	control	6	2420	61 days	4.5 °C	Yes	Yes	1948	2353
172	1	*Gasterosteus aculeatus*	6	1189	91 days	1.5 °C	Yes	No	725	NA
173	4	*Notonecta* sp.	6	1091	61 days	4.5 °C	Yes	Yes	828	1141
174	1	*Aeshna cyanea*	6	832	61 days	1.5 °C	Yes	No	524	NA

175	5	*Aeshna cyanea*	6	1164	61 days	1.5 °C	Yes	Yes	886	1121
176	9	*Gasterosteus aculeatus*	6	1694	61 days	4.5 °C	Yes	Yes	1447	1737
177	2	*Aeshna cyanea*	6	1061	91 days	1.5 °C	Yes	No	650	NA
178	8	*Aeshna cyanea*	6	1177	61 days	4.5 °C	Yes	Yes	1096	1522
179	6	*Gasterosteus aculeatus*	6	1038	61 days	1.5 °C	Yes	Yes	858	934
180	9	control	6	2168	61 days	1.5 °C	Yes	Yes	2014	2313
181	10	*Notonecta* sp.	6	1129	91 days	1.5 °C	Yes	Yes	720	712
182	8	*Lissotriton vulgaris*	6	1478	61 days	4.5 °C	Yes	Yes	1365	1548
183	1	*Lissotriton vulgaris*	6	1502	61 days	1.5 °C	Yes	Yes	1352	1499
184	7	*Lissotriton vulgaris*	6	1032	61 days	4.5 °C	Yes	Yes	908	1203
185	9	*Lissotriton vulgaris*	6	1851	61 days	1.5 °C	Yes	Yes	1696	1648
186	9	*Aeshna cyanea*	6	1447	61 days	1.5 °C	Yes	Yes	1222	1343
187	2	*Notonecta* sp.	6	1028	61 days	4.5 °C	Yes	Yes	846	877
188	3	*Lissotriton vulgaris*	6	826	91 days	1.5 °C	No	NA	NA	NA
189	7	*Notonecta* sp.	6	1184	91 days	4.5 °C	Yes	Yes	907	1055
190	2	*Lissotriton vulgaris*	6	974	91 days	1.5 °C	Yes	Yes	767	890
191	8	*Notonecta* sp.	8	1015	61 days	1.5 °C	Yes	No	629	NA
192	6	*Aeshna cyanea*	8	706	91 days	4.5 °C	Yes	No	463	NA
193	8	*Gasterosteus aculeatus*	8	1069	91 days	4.5 °C	Yes	Yes	792	686
194	2	*Notonecta* sp.	8	834	91 days	1.5 °C	No	NA	NA	NA
195	3	*Notonecta* sp.	8	683	91 days	1.5 °C	No	NA	NA	NA
196	7	*Gasterosteus aculeatus*	8	748	91 days	4.5 °C	Yes	Yes	552	597
197	3	*Aeshna cyanea*	8	640	61 days	4.5 °C	Yes	Yes	430	530
198	9	*Lissotriton vulgaris*	8	634	91 days	4.5 °C	Yes	No	378	NA
199	3	*Lissotriton vulgaris*	8	728	61 days	1.5 °C	No	NA	NA	NA
200	10	*Notonecta* sp.	8	1170	61 days	1.5 °C	Yes	Yes	1261	1383
201	2	*Lissotriton vulgaris*	8	770	91 days	4.5 °C	Yes	Yes	486	505
202	9	*Aeshna cyanea*	8	1270	91 days	4.5 °C	Yes	Yes	968	1106

203	10	*Aeshna cyanea*	8	1635	61 days	1.5 °C	Yes	Yes	1376	1713
204	7	*Notonecta* sp.	8	1171	61 days	4.5 °C	Yes	Yes	988	1316
205	1	*Notonecta* sp.	8	1141	61 days	1.5 °C	Yes	Yes	1054	1104
206	8	*Lissotriton vulgaris*	8	902	91 days	1.5 °C	Yes	No	525	NA
207	1	control	8	1520	61 days	1.5 °C	Yes	Yes	1399	1629
208	1	*Gasterosteus aculeatus*	8	1182	61 days	4.5 °C	Yes	Yes	1128	1152
209	5	control	8	864	61 days	4.5 °C	Yes	Yes	694	761
210	7	*Aeshna cyanea*	8	883	61 days	4.5 °C	Yes	Yes	665	737
211	7	*Lissotriton vulgaris*	8	988	61 days	1.5 °C	Yes	No	591	NA
212	2	*Gasterosteus aculeatus*	8	939	91 days	1.5 °C	Yes	No	548	NA
213	10	*Gasterosteus aculeatus*	8	1391	91 days	4.5 °C	Yes	Yes	1292	1212
214	4	*Gasterosteus aculeatus*	8	706	91 days	1.5 °C	Yes	Yes	599	481
215	4	*Lissotriton vulgaris*	8	563	61 days	1.5 °C	Yes	No	421	NA
216	6	*Lissotriton vulgaris*	8	793	61 days	1.5 °C	Yes	Yes	672	624
217	7	control	8	996	61 days	1.5 °C	Yes	Yes	707	733
218	8	*Aeshna cyanea*	8	928	91 days	1.5 °C	No	NA	NA	NA
219	9	control	8	801	61 days	1.5 °C	Yes	Yes	742	858
220	5	*Gasterosteus aculeatus*	8	1054	91 days	4.5 °C	Yes	No	502	NA
221	4	control	8	811	91 days	4.5 °C	Yes	Yes	680	698
222	2	control	8	1144	61 days	4.5 °C	Yes	Yes	1126	1164
223	5	*Aeshna cyanea*	8	1140	91 days	4.5 °C	Yes	Yes	894	1059
224	10	*Lissotriton vulgaris*	8	1048	61 days	4.5 °C	Yes	Yes	863	1126
225	6	*Gasterosteus aculeatus*	8	556	91 days	4.5 °C	No	NA	NA	NA
226	2	*Aeshna cyanea*	8	834	61 days	4.5 °C	Yes	Yes	649	1044
227	1	*Lissotriton vulgaris*	8	1195	61 days	4.5 °C	Yes	Yes	1050	1266
228	5	*Lissotriton vulgaris*	8	754	91 days	4.5 °C	No	NA	NA	NA
229	1	*Aeshna cyanea*	8	1456	91 days	4.5 °C	Yes	No	805	NA
230	9	*Gasterosteus aculeatus*	8	999	61 days	1.5 °C	Yes	Yes	703	869

231	4	*Notonecta* sp.	8	654	91 days	1.5 °C	No	NA	NA	NA
232	10	control	8	1716	61 days	1.5 °C	Yes	Yes	1212	1344
233	9	*Notonecta* sp.	8	783	91 days	1.5 °C	Yes	Yes	616	648
234	7	*Aeshna cyanea*	12	1127	91 days	4.5 °C	Yes	Yes	821	785
235	10	*Gasterosteus aculeatus*	12	1488	91 days	1.5 °C	No	NA	NA	NA
236	1	*Notonecta* sp.	12	1380	61 days	1.5 °C	Yes	Yes	1179	1205
237	1	control	12	1229	91 days	4.5 °C	Yes	Yes	868	889
238	1	*Aeshna cyanea*	12	1215	61 days	1.5 °C	Yes	Yes	951	1203
239	8	*Aeshna cyanea*	12	1456	61 days	1.5 °C	Yes	Yes	990	1309
240	4	*Gasterosteus aculeatus*	12	786	91 days	4.5 °C	Yes	Yes	771	644
241	2	*Aeshna cyanea*	12	1198	61 days	4.5 °C	Yes	Yes	952	978
242	3	*Notonecta* sp.	12	826	61 days	1.5 °C	Yes	Yes	820	826
243	5	*Gasterosteus aculeatus*	12	1003	91 days	1.5 °C	No	NA	NA	NA
244	10	control	12	1259	61 days	4.5 °C	Yes	Yes	897	1185
245	9	*Notonecta* sp.	12	1349	61 days	4.5 °C	Yes	Yes	1248	1276
246	7	*Lissotriton vulgaris*	12	1283	61 days	4.5 °C	Yes	Yes	1076	1089
247	4	*Notonecta* sp.	12	1103	91 days	4.5 °C	Yes	Yes	920	615
248	9	control	12	1132	61 days	4.5 °C	Yes	Yes	1045	1039
249	2	control	12	1390	61 days	4.5 °C	Yes	Yes	1181	1239
250	8	*Lissotriton vulgaris*	12	1194	91 days	1.5 °C	No	NA	NA	NA
251	8	*Notonecta* sp.	12	901	91 days	4.5 °C	Yes	Yes	781	615
252	9	*Notonecta* sp.	12	1558	91 days	1.5 °C	Yes	Yes	1221	1262
253	3	*Lissotriton vulgaris*	12	910	61 days	4.5 °C	Yes	Yes	891	981
254	10	*Lissotriton vulgaris*	12	1108	61 days	1.5 °C	Yes	Yes	1025	988
255	1	*Gasterosteus aculeatus*	12	1559	91 days	1.5 °C	Yes	Yes	1287	1366
256	4	*Lissotriton vulgaris*	12	947	61 days	4.5 °C	Yes	Yes	799	753
257	1	*Lissotriton vulgaris*	12	1323	91 days	4.5 °C	Yes	Yes	784	858
258	7	*Gasterosteus aculeatus*	12	1062	61 days	1.5 °C	Yes	Yes	1120	1111

259	6	control	12	922	91 days	1.5 °C	Yes	Yes	659	491
260	5	control	12	962	91 days	4.5 °C	No	NA	NA	NA
261	7	control	12	1179	91 days	1.5 °C	Yes	Yes	965	1029
262	2	*Lissotriton vulgaris*	12	1412	61 days	1.5 °C	Yes	Yes	1411	1467
263	4	control	12	1485	61 days	1.5 °C	Yes	Yes	1402	1692
264	5	*Lissotriton vulgaris*	12	1144	91 days	4.5 °C	Yes	Yes	747	800
265	6	*Aeshna cyanea*	12	1264	91 days	1.5 °C	Yes	Yes	1087	1246
266	8	*Lissotriton vulgaris*	7	1023	61 days	4.5 °C	Yes	Yes	804	1033
267	10	*Lissotriton vulgaris*	7	1145	91 days	1.5 °C	Yes	Yes	827	1027
268	1	control	7	1918	91 days	1.5 °C	Yes	Yes	1487	1470
269	1	*Lissotriton vulgaris*	7	1354	91 days	1.5 °C	Yes	Yes	1090	1251
270	4	*Aeshna cyanea*	7	1172	61 days	1.5 °C	Yes	Yes	1209	1329
271	9	*Aeshna cyanea*	7	724	91 days	1.5 °C	No	NA	NA	NA
272	2	*Lissotriton vulgaris*	7	1191	91 days	4.5 °C	Yes	Yes	969	702
273	10	*Aeshna cyanea*	7	1928	91 days	1.5 °C	Yes	Yes	1568	1112
274	7	*Aeshna cyanea*	7	1638	61 days	1.5 °C	Yes	Yes	1477	1585
275	8	*Notonecta* sp.	7	1266	91 days	1.5 °C	Yes	Yes	1112	698
276	9	*Gasterosteus aculeatus*	7	1966	61 days	1.5 °C	Yes	Yes	1778	1898
277	7	control	7	1617	91 days	1.5 °C	Yes	Yes	1530	1384
278	3	*Notonecta* sp.	7	1343	61 days	1.5 °C	Yes	Yes	1209	1433
279	4	*Notonecta* sp.	7	1062	91 days	4.5 °C	Yes	Yes	779	834
280	2	control	7	1095	61 days	1.5 °C	Yes	No	895	NA
281	9	*Lissotriton vulgaris*	7	2080	91 days	1.5 °C	Yes	Yes	1944	1648
282	2	*Aeshna cyanea*	7	1113	61 days	1.5 °C	Yes	Yes	1170	1372
283	3	*Aeshna cyanea*	7	967	91 days	4.5 °C	Yes	Yes	691	682
284	10	*Gasterosteus aculeatus*	7	1906	91 days	4.5 °C	Yes	Yes	1543	1456
285	5	*Gasterosteus aculeatus*	7	785	91 days	4.5 °C	No	NA	NA	NA
286	5	*Lissotriton vulgaris*	7	1033	61 days	1.5 °C	Yes	Yes	820	1052

287	9	*Notonecta* sp.	7	1710	61 days	1.5 °C	Yes	Yes	1489	1667
288	9	control	7	1797	61 days	1.5 °C	Yes	Yes	1678	1884
289	6	*Lissotriton vulgaris*	7	1154	61 days	4.5 °C	Yes	Yes	859	992
290	8	*Aeshna cyanea*	7	1089	91 days	1.5 °C	Yes	Yes	833	994
291	8	control	7	929	61 days	1.5 °C	No	NA	NA	NA
292	8	*Notonecta* sp.	7	1360	61 days	1.5 °C	Yes	Yes	1195	1258
293	7	*Lissotriton vulgaris*	7	1199	91 days	4.5 °C	Yes	Yes	824	658
294	4	control	7	1122	91 days	1.5 °C	Yes	Yes	770	841
295	1	*Aeshna cyanea*	7	1521	61 days	1.5 °C	Yes	Yes	1201	1430
296	10	control	7	2187	91 days	4.5 °C	Yes	Yes	1990	1986
298	7	*Notonecta* sp.	7	1521	91 days	4.5 °C	Yes	No	885	NA
299	1	*Notonecta* sp.	7	2015	61 days	4.5 °C	Yes	No	1293	NA
300	3	control	11	849	91 days	4.5 °C	Yes	Yes	657	718
301	9	*Gasterosteus aculeatus*	11	1425	61 days	4.5 °C	Yes	Yes	1227	1819
302	10	control	11	1958	91 days	1.5 °C	Yes	Yes	1597	1415
303	8	control	11	897	91 days	4.5 °C	Yes	Yes	887	979
304	1	*Lissotriton vulgaris*	11	1644	91 days	4.5 °C	Yes	Yes	1426	1528
305	8	*Notonecta* sp.	11	949	61 days	4.5 °C	Yes	Yes	868	1011
306	2	*Notonecta* sp.	11	1104	61 days	1.5 °C	No	NA	NA	NA
307	8	*Aeshna cyanea*	11	1259	91 days	1.5 °C	Yes	No	718	NA
308	9	*Aeshna cyanea*	11	2472	61 days	4.5 °C	Yes	Yes	1952	2890
309	1	*Notonecta* sp.	11	986	61 days	4.5 °C	Yes	Yes	876	1116
310	8	*Lissotriton vulgaris*	11	1870	61 days	1.5 °C	Yes	Yes	1598	1809
311	5	*Aeshna cyanea*	11	1351	61 days	4.5 °C	Yes	Yes	1040	1221
312	8	*Gasterosteus aculeatus*	11	1309	61 days	1.5 °C	Yes	Yes	985	1176
313	2	control	11	1434	91 days	4.5 °C	Yes	Yes	1263	1333
314	5	*Gasterosteus aculeatus*	11	1651	61 days	4.5 °C	Yes	Yes	1349	1569
315	7	*Lissotriton vulgaris*	11	1487	91 days	1.5 °C	No	NA	NA	NA

316	10	*Lissotriton vulgaris*	11	1682	61 days	4.5 °C	Yes	Yes	1626	1933
317	3	*Gasterosteus aculeatus*	11	1299	61 days	4.5 °C	Yes	Yes	1082	952
318	7	*Aeshna cyanea*	11	1468	61 days	1.5 °C	Yes	Yes	1064	1282
319	10	*Gasterosteus aculeatus*	11	831	61 days	4.5 °C	Yes	Yes	759	1032
320	10	*Notonecta* sp.	11	2205	91 days	1.5 °C	Yes	Yes	1670	1364
321	10	*Aeshna cyanea*	11	1611	91 days	4.5 °C	Yes	Yes	1415	1192
322	3	*Notonecta* sp.	11	837	61 days	4.5 °C	Yes	Yes	668	867
323	2	*Lissotriton vulgaris*	11	1275	61 days	4.5 °C	Yes	Yes	1239	1638
324	6	*Lissotriton vulgaris*	11	1237	61 days	1.5 °C	Yes	Yes	1220	1416
325	7	*Gasterosteus aculeatus*	11	1573	91 days	4.5 °C	No	NA	NA	NA
326	1	*Aeshna cyanea*	11	1458	61 days	4.5 °C	Yes	Yes	1214	1177
327	9	*Lissotriton vulgaris*	11	2360	61 days	4.5 °C	Yes	Yes	2100	2099
328	6	*Aeshna cyanea*	11	863	61 days	1.5 °C	Yes	Yes	640	679
329	5	*Lissotriton vulgaris*	11	1283	91 days	1.5 °C	No	NA	NA	NA
330	9	control	11	1555	61 days	4.5 °C	Yes	Yes	1366	1600
331	10	*Lissotriton vulgaris*	9	1263	91 days	4.5 °C	Yes	Yes	911	1017
332	2	*Aeshna cyanea*	9	860	91 days	4.5 °C	Yes	Yes	695	899
333	8	control	9	601	91 days	1.5 °C	No	NA	NA	NA
334	3	*Lissotriton vulgaris*	9	819	91 days	4.5 °C	Yes	Yes	667	426
335	4	*Aeshna cyanea*	9	805	91 days	1.5 °C	Yes	Yes	889	779
336	1	*Aeshna cyanea*	9	783	61 days	4.5 °C	Yes	Yes	631	565
337	1	control	9	754	61 days	1.5 °C	Yes	Yes	627	738
338	2	control	9	707	91 days	1.5 °C	Yes	Yes	750	577
339	5	*Aeshna cyanea*	9	950	91 days	1.5 °C	No	NA	NA	NA
340	9	*Notonecta* sp.	9	1343	91 days	1.5 °C	Yes	Yes	1095	1352
341	8	*Gasterosteus aculeatus*	9	553	61 days	1.5 °C	No	NA	NA	NA
342	10	*Notonecta* sp.	9	1897	91 days	1.5 °C	No	NA	NA	NA
343	2	*Lissotriton vulgaris*	9	965	61 days	1.5 °C	Yes	Yes	873	1006

344	4	*Gasterosteus aculeatus*	9	755	61 days	1.5 °C	Yes	Yes	774	941
345	6	control	9	481	61 days	4.5 °C	No	NA	NA	NA
346	9	control	9	1352	61 days	4.5 °C	Yes	Yes	1103	1207
347	6	*Lissotriton vulgaris*	9	530	91 days	1.5 °C	No	NA	NA	NA
348	4	*Notonecta* sp.	9	696	61 days	1.5 °C	Yes	Yes	675	858
349	10	control	9	1325	91 days	1.5 °C	Yes	Yes	745	918
350	9	*Lissotriton vulgaris*	9	1085	91 days	1.5 °C	No	NA	NA	NA
351	2	*Gasterosteus aculeatus*	9	740	61 days	4.5 °C	Yes	Yes	710	773
352	1	*Lissotriton vulgaris*	9	1100	91 days	4.5 °C	Yes	Yes	921	782
353	3	control	9	743	91 days	1.5 °C	No	NA	NA	NA
354	10	*Aeshna cyanea*	9	803	91 days	4.5 °C	Yes	Yes	704	622
355	9	*Aeshna cyanea*	9	1078	91 days	4.5 °C	Yes	Yes	929	863
356	5	*Gasterosteus aculeatus*	9	784	61 days	4.5 °C	Yes	Yes	702	873
357	8	*Lissotriton vulgaris*	9	1107	61 days	1.5 °C	Yes	Yes	947	975
358	4	control	9	821	61 days	1.5 °C	Yes	Yes	571	532
359	7	*Lissotriton vulgaris*	9	596	91 days	4.5 °C	Yes	Yes	542	432
360	6	*Gasterosteus aculeatus*	9	812	61 days	1.5 °C	Yes	Yes	701	943
361	5	control	9	641	61 days	1.5 °C	Yes	No	414	NA
362	3	*Notonecta* sp.	9	831	91 days	4.5 °C	Yes	Yes	683	537
363	7	*Gasterosteus aculeatus*	9	961	61 days	4.5 °C	Yes	Yes	851	976
364	10	*Gasterosteus aculeatus*	9	1278	61 days	1.5 °C	Yes	No	831	NA
375	7	*Aeshna cyanea*	10	1157	91 days	1.5 °C	Yes	No	634	NA
376	5	control	10	1225	61 days	1.5 °C	No	NA	NA	NA
377	1	*Gasterosteus aculeatus*	10	1213	91 days	4.5 °C	Yes	Yes	1055	1194
~~378~~	4	*Lissotriton vulgaris*	10	1467	61 days	1.5 °C	NA	NA	NA	NA
379	6	*Notonecta* sp.	10	965	91 days	4.5 °C	Yes	Yes	804	675
380	7	*Lissotriton vulgaris*	10	1634	91 days	4.5 °C	Yes	Yes	1467	1655
381	7	control	10	968	61 days	4.5 °C	Yes	Yes	937	1068

382	6	*Lissotriton vulgaris*	10	1228	91 days	4.5 °C	Yes	Yes	1052	1140
383	10	control	10	1791	61 days	4.5 °C	Yes	Yes	1644	2226
384	9	control	10	1979	91 days	4.5 °C	Yes	Yes	1585	1635
385	4	*Notonecta* sp.	10	1204	91 days	1.5 °C	Yes	Yes	914	609
386	2	*Gasterosteus aculeatus*	10	1554	91 days	4.5 °C	Yes	Yes	1459	1422
387	8	*Lissotriton vulgaris*	10	1577	61 days	4.5 °C	Yes	Yes	1573	1694
388	9	*Aeshna cyanea*	10	3279	91 days	4.5 °C	Yes	Yes	3138	3393
389	2	*Notonecta* sp.	10	1124	91 days	4.5 °C	Yes	Yes	962	951
390	10	*Aeshna cyanea*	10	1201	91 days	1.5 °C	Yes	Yes	836	673
391	1	control	10	1297	91 days	4.5 °C	Yes	Yes	1296	1089
392	6	*Notonecta* sp.	10	1364	91 days	4.5 °C	Yes	Yes	1207	1026
393	8	*Gasterosteus aculeatus*	10	1226	91 days	1.5 °C	Yes	Yes	751	855
394	8	*Aeshna cyanea*	10	1347	61 days	4.5 °C	Yes	Yes	1233	1586
395	4	control	10	1016	61 days	4.5 °C	Yes	Yes	972	1300
396	9	*Notonecta* sp.	10	1250	61 days	4.5 °C	Yes	Yes	1051	1321

Table S5. Temperature regimes experienced by juvenile common toads in different hibernation scenarios. Temperatures were obtained by placing three data loggers each in two laboratory refrigerators set to 1.5 and 4.5 °C, respectively. Means ± s.d. are presented.

Hibernation scenario	Temperature (°C) Data logger 1	Data logger 2	Data logger 3	Mean
1.5 °C, 91 days	0.863 ± 0.535	1.202 ± 0.446	1.822 ± 0.707	1.306 ± 0.719
1.5 °C, 61 days	0.942 ± 0.421	1.211 ± 0.359	2.203 ± 0.380	1.452 ± 0.667
4.5 °C, 91 days	3.74 ± 0.409	4.469 ± 0.309	4.824 ± 0.318	4.291 ± 0.567
4.5 °C, 61 days	3.822 ± 0.342	4.488 ± 0.289	4.846 ± 0.294	4.385 ± 0.525

Table S6. Effects of the three-way interaction of hibernation temperature, hibernation length and mass before hibernation. Results were obtained from type III analysis-of-deviance tables with Wald χ^2 tests using the car package in R. Terms including larval environment could not be estimated because in some subgroups there was no variance (all individuals survived), but corresponding graphs did not suggest significant effects of larval environment. Graphs are available from the authors upon request.

	N	χ^2	df	*P*
<u>Survival after hibernation</u>				
intercept	325	3.42	1	0.065
hibernation length		0.23	1	0.629
hibernation temperature		8.84	1	0.003
mass before hibernation		4.38	1	0.037
hibernation length × hibernation temperature		4.43	1	0.035
hibernation length × mass before hibernation		0.28	1	0.596
hibernation temperature × mass before hibernation		8.71	1	0.003
hibernation length × hibernation temperature × mass before hibernation		4.35	1	0.037

Table S7. Main effects and interactions in analyses of survival and body mass change after hibernation. Non-significant effects were calculated by including each effect separately into the final models (containing only the significant effects, marked with asterisk). Results were obtained from type III analysis-of-deviance tables with Wald χ^2 tests using the car package in R. In case of survival, terms including larval environment could not be estimated because in some subgroups there was no variance (all individuals survived), but corresponding graphs did not suggest significant effects of larval environment. Graphs are available from the authors upon request.

	N	χ^2	df	*P*
Survival after hibernation				
intercept	325	41.56	1	<0.001
hibernation length		2.76	1	0.097
hibernation temperature *		4.47	1	0.035
hibernation length × hibernation temperature		0.29	1	0.592
Body mass change after hibernation				
intercept	297	39.29	1	<0.001
hibernation length *		28.71	1	<0.001
hibernation temperature *		4.91	1	0.027
larval environment		9.36	4	0.053
mass before hibernation		0.30	1	0.586
hibernation length × hibernation temperature *		4.96	1	0.026
hibernation length × larval environment		2.42	4	0.659
hibernation length × mass before hibernation		1.25	1	0.265
hibernation temperature × larval environment		1.03	4	0.906
hibernation temperature × mass before hibernation		0.10	1	0.756
larval environment × mass before hibernation		1.20	4	0.879
hibernation length × hibernation temperature × mass before hibernation		0.20	1	0.653
hibernation length × larval environment × mass before hibernation		5.40	4	0.249
hibernation temperature × larval environment × mass before hibernation		2.70	4	0.609
larval environment × hibernation length × hibernation temperature		5.96	4	0.202

Figure S1. Effects of larval environment on the survival of toadlets during hibernation (N=371). Abbreviations as follows: C: control (no predators), N: *Notonecta* sp., A: *Aeshna cyanea*, G: *Gasterosteus aculeatus*, L: *Lissotriton vulgaris*.

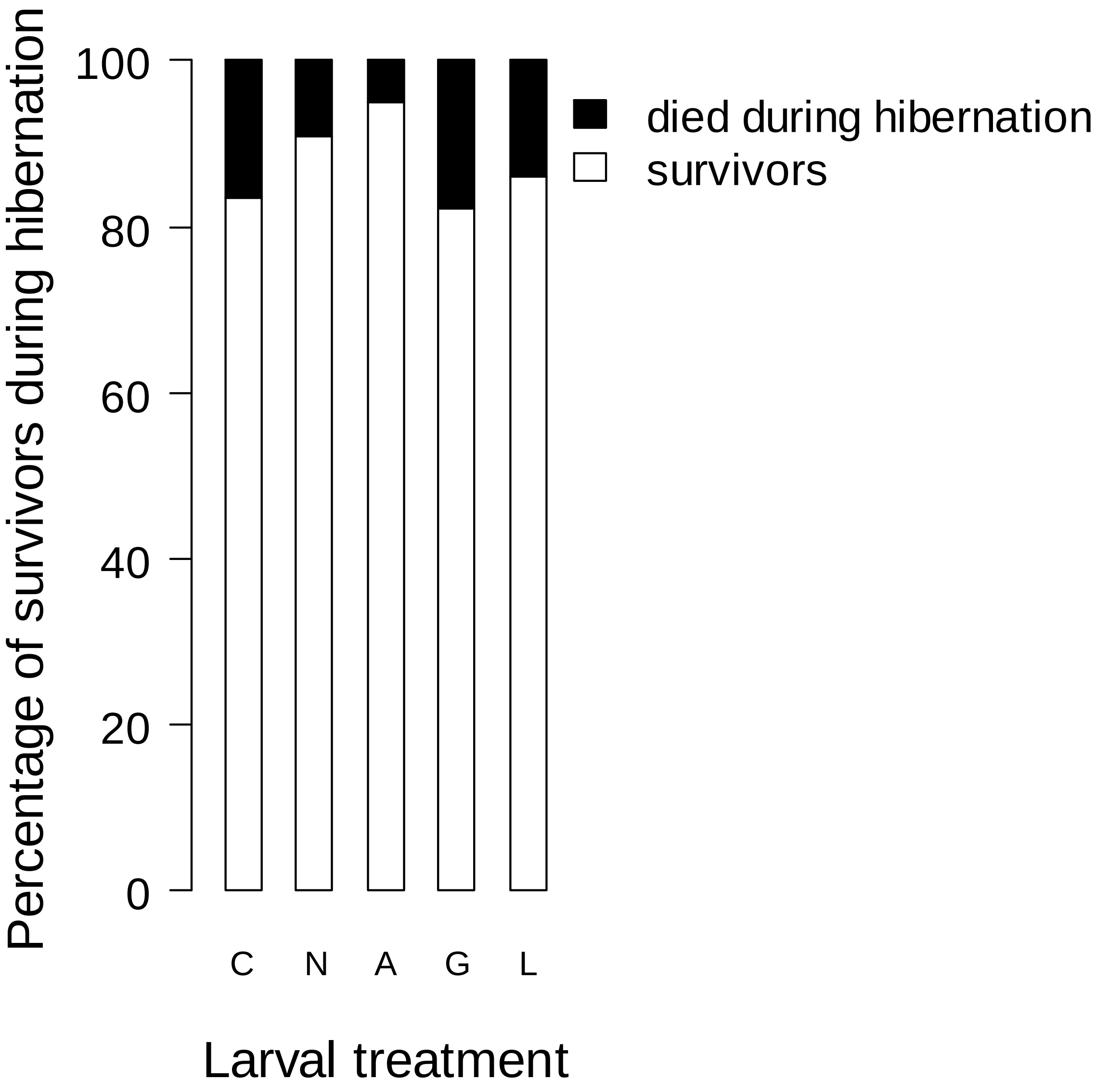

Figure S2. Numbers of surviving and dead toadlets during and after hibernation in our experiment.

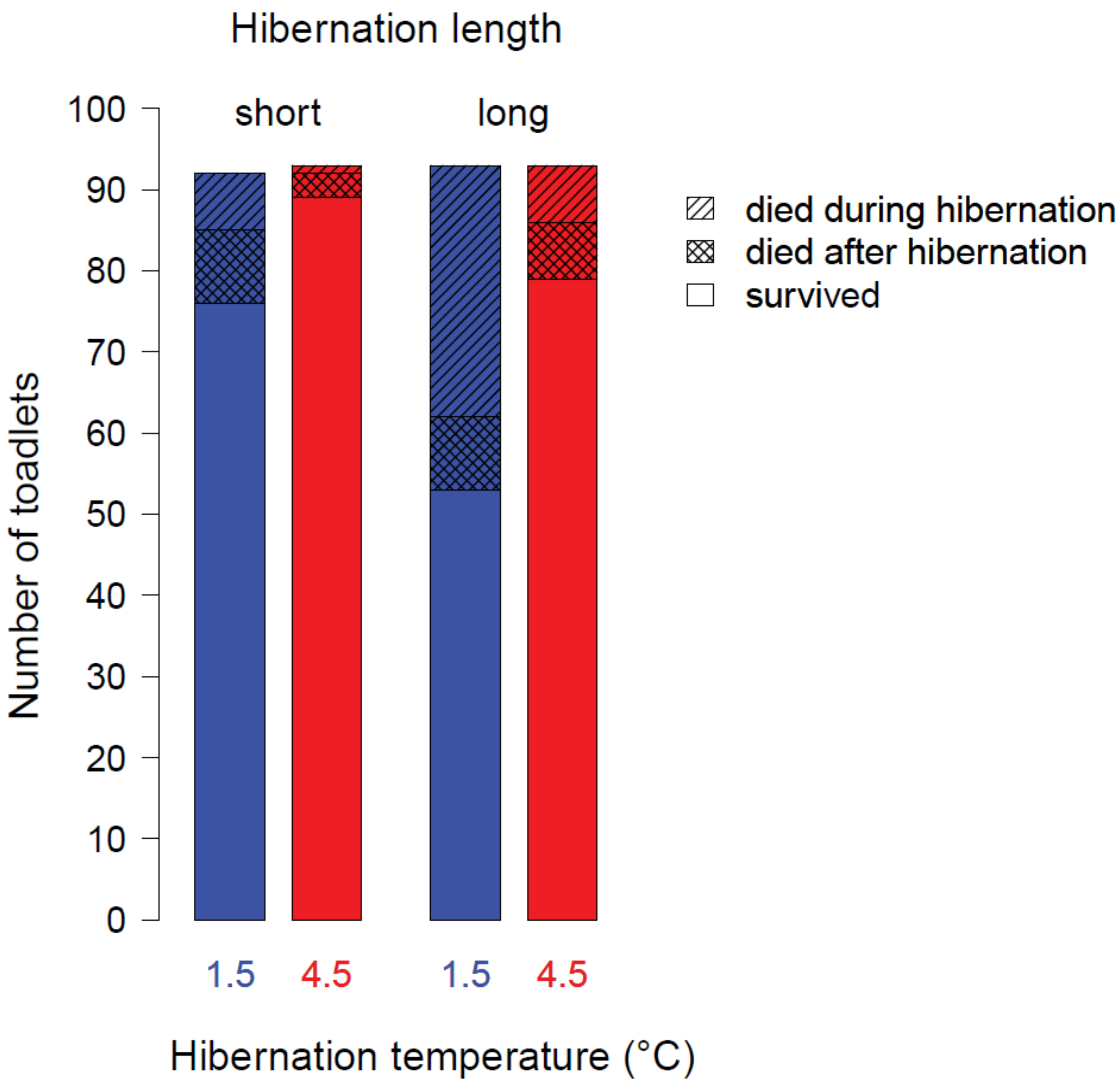

Figure S3. Pre-hibernation mass and sample size of toadlets that died or survived after hibernation. A significant three-way interaction effect on survival after hibernation was caused by a few large dead toadlets in the 4.5 °C, short hibernation scenario that had experienced large mass loss during hibernation. Sample sizes are presented above error bars.

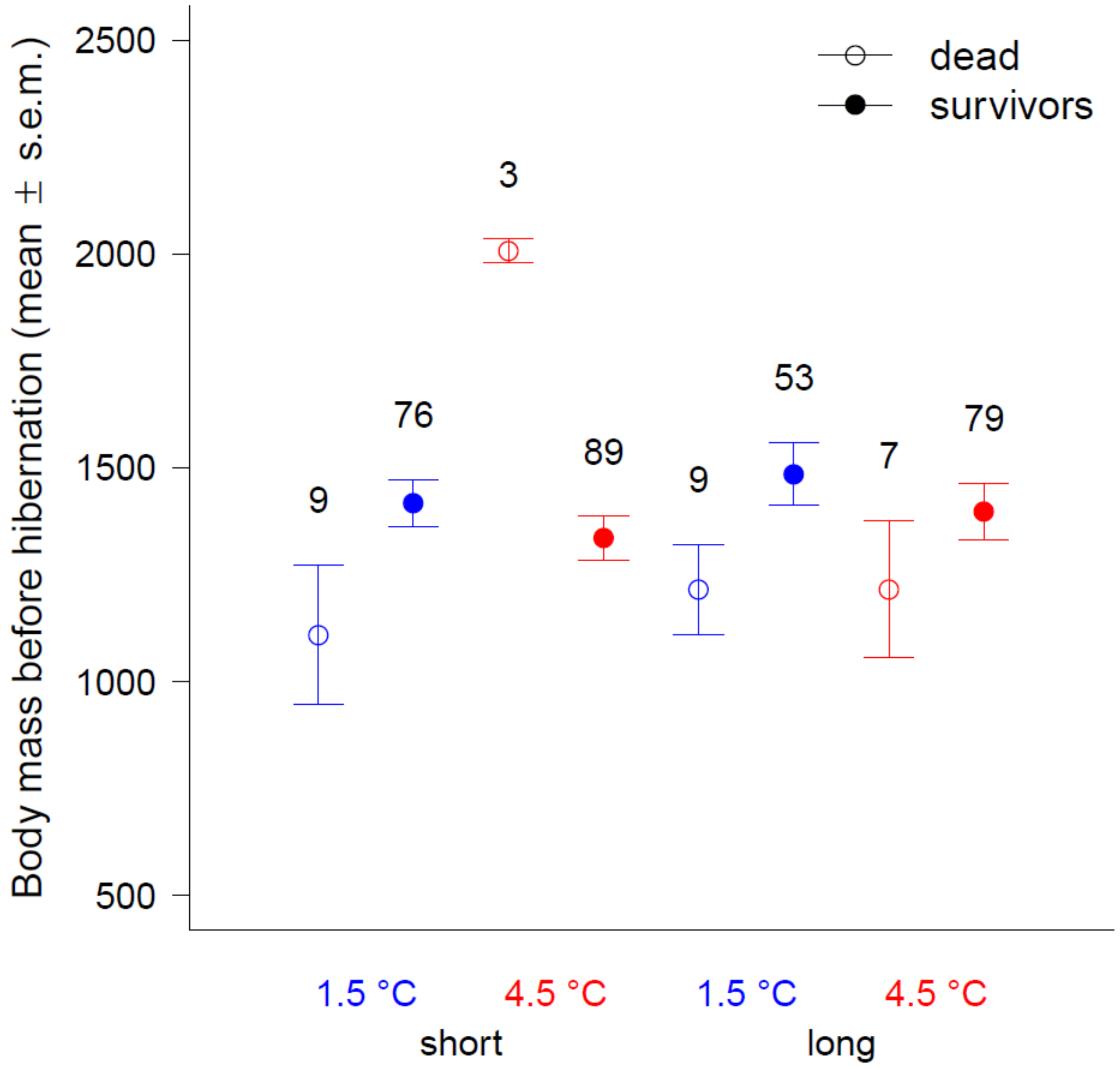

Figure S4. Body mass change of toadlets between two consecutive weighing occasions after hibernation. Body mass change was calculated as mass three weeks after hibernation/mass one week after hibernation. Values larger than 1 indicate body mass gain. Letters above error bars represent pairwise comparisons; groups marked with different letters differ significantly based on linear contrasts corrected for false discovery rate.

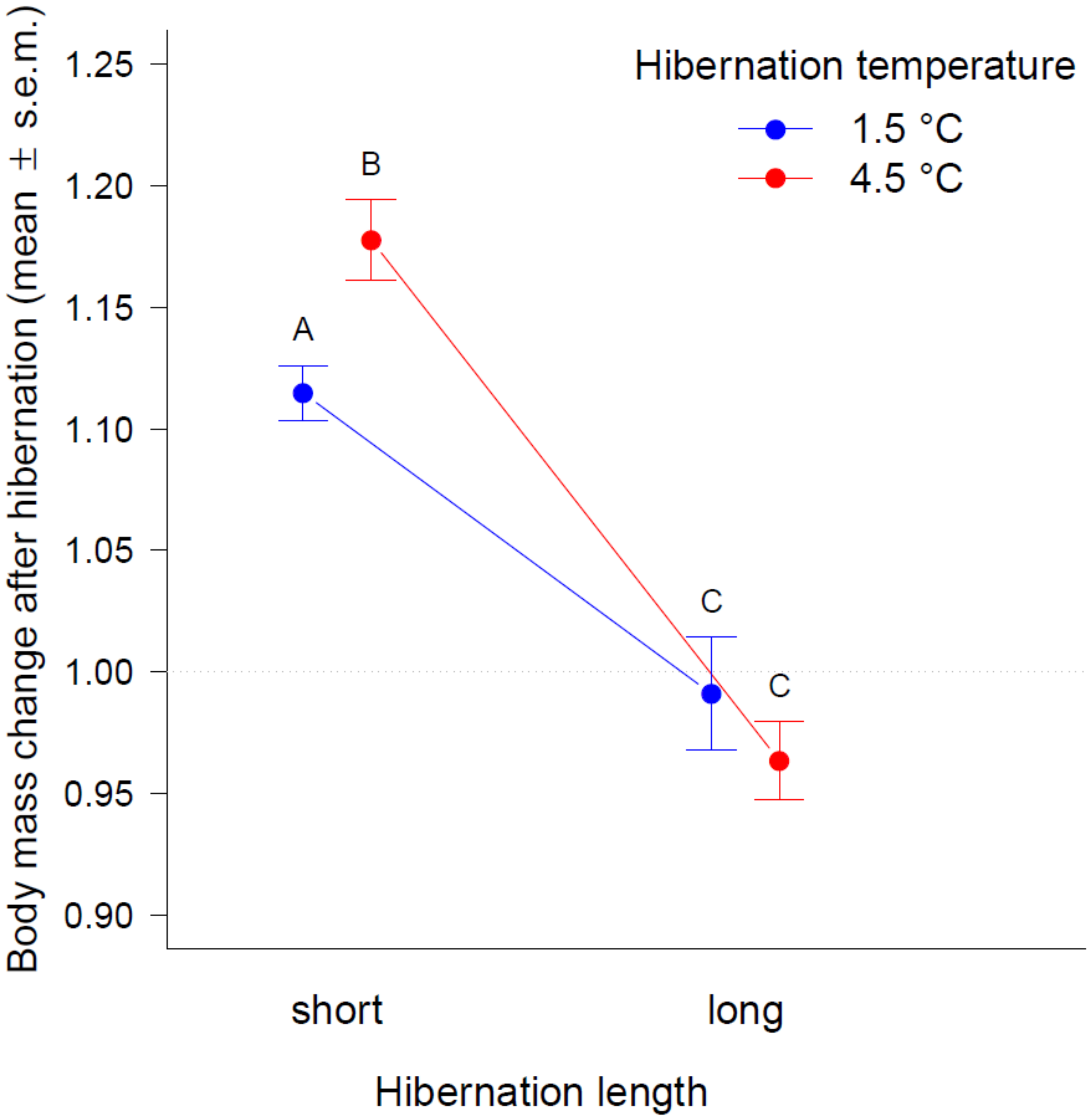

Figure S5. Body mass change of toadlets from different larval environments between two consecutive weighing occasions after hibernation. Body mass change was calculated as mass three weeks after hibernation/mass one week after hibernation. Values above 1 indicate body mass gain. Abbreviations as follows: C: control (no predators), N: *Notonecta* sp., A: *Aeshna cyanea*, G: *Gasterosteus aculeatus*, L: *Lissotriton vulgaris*.

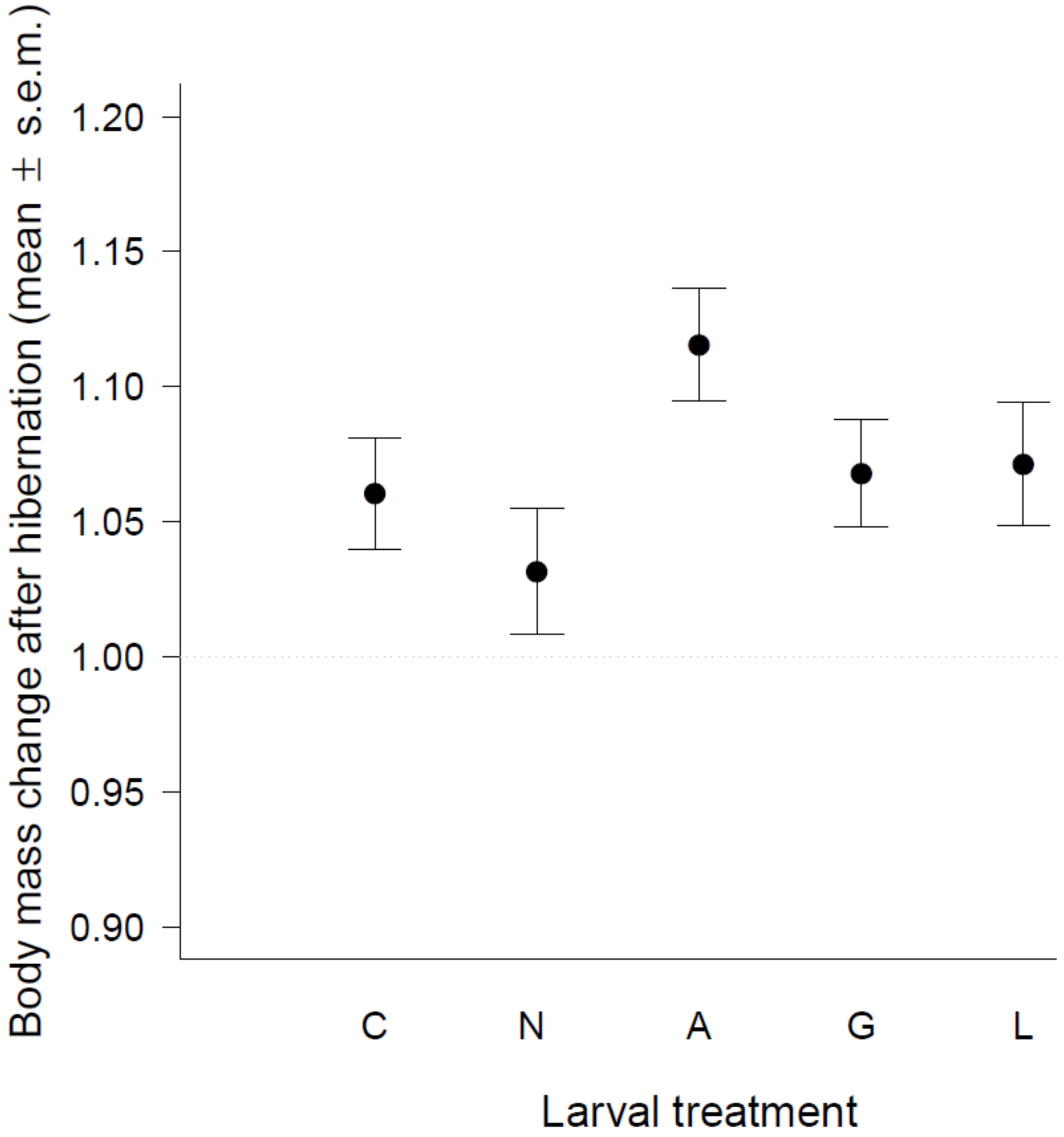

Supplement: Supplementary Information [file srep26754-s1.pdf]
